# Increased expression of colony stimulating factor-1 is a predictor of poor prognosis in patients with clear-cell renal cell carcinoma

**DOI:** 10.1186/s12885-015-1076-5

**Published:** 2015-02-18

**Authors:** Liu Yang, Qian Wu, Le Xu, Weijuan Zhang, Yu Zhu, Haiou Liu, Jiejie Xu, Jianxin Gu

**Affiliations:** 1 Key Laboratory of Glycoconjugate Research, MOH, Department of Biochemistry and Molecular Biology, School of Basic Medical Sciences, Shanghai Medical College of Fudan University, Mailbox 103, 138 Yixueyuan Road, Shanghai, 200032 China; 2 Department of Urology, Zhongshan Hospital, Fudan University, Shanghai, 200032 China; 3 Department of Immunology, School of Basic Medical Sciences, Shanghai Medical College of Fudan University, Shanghai, 200032 China; 4 Department of Urology, Ninth People’s Hospital, School of Medicine, Shanghai Jiaotong University, Shanghai, 200011 China

**Keywords:** Clear-cell renal cell carcinoma, Colony stimulating factor-1, Prognostic biomarker, Recurrence-free survival, Cancer-specific survival

## Abstract

**Background:**

This study aims to evaluate the impact of colony stimulating factor-1 (CSF-1) expression on recurrence and survival of patients with clear-cell renal cell carcinoma (ccRCC) following surgery.

**Methods:**

We retrospectively enrolled 267 patients (195 in the training cohort and 72 in the validation cohort) with ccRCC undergoing nephrectomy at a single institution. Clinicopathologic features, cancer-specific survival (CSS) and recurrence-free survival (RFS) were recorded. CSF-1 levels were assessed by immunohistochemistry in tumor tissues. Kaplan-Meier method was applied to compare survival curves. Cox regression models were used to analyze the impact of prognostic factors on CSS and RFS. Concordance index (C-index) was calculated to assess predictive accuracy.

**Results:**

In both cohorts, CSF-1 expression positively correlated with advanced Fuhrman grade and necrosis. High CSF-1 expression indicated poor survival and early recurrence of ccRCC patients after surgery, especially those with advanced TNM stage disease. Multivariate Cox regression analysis showed CSF-1 expression was an independent unfavorable prognostic factor for recurrence and survival. The predictive accuracy of the University of California Los Angeles Integrated Staging System (UISS) was significantly improved when CSF-1 expression was incorporated.

**Conclusions:**

High CSF-1 expression is a potential adverse prognostic biomarker for recurrence and survival of ccRCC patients after nephrectomy.

## Background

Renal cell carcinoma (RCC) accounts for approximately 3% of all adult malignancies, representing the seventh most common cancer in men and the ninth most common cancer in women. Based on current guidelines, surgery remains the only curative treatment option in patients with localized renal cell carcinoma (RCC) [1-3]. However, despite the durable long-term disease control in most patients, about 30% of patients with localized disease experience local recurrence or distant metastasis after adequately performed nephrectomy. Currently, several prognostic models have been proposed to identify patients at a high risk of disease progression after nephrectomy. The two commonly used models are UISS [4] and Mayo stage, size, grade, and necrosis score (SSIGN) score [5]. The predictive accuracy of these models may be further improved by the incorporation of novel prognostic biomarkers.

Colony stimulating factor-1 (CSF-1), also known as macrophage colony-stimulating factor (M-CSF), is the primary cytokine that regulates the proliferation and differentiation of monocytes and macrophages [6]. CSF-1 is secreted by various types of cells like monocytes, fibroblasts, endothelial cells, and tumor cells. All the biological effects of CSF-1 are mediated through CSF-1 receptor (CSF-1R), a receptor belonging to type III receptor tyrosine kinase family. Many studies have demonstrated that CSF-1 can polarize macrophages in the tumor microenvironment to an M2 phenotype, which has anti-inflammatory function, favors angiogenesis, and promotes tumor growth [7-9]. Moreover, recent evidences have revealed that the infiltration of M2 macrophages is closely associated with unfavorable prognosis in many types of cancer [10-18].

In this study, we analyzed CSF-1 expression by immunohistochemistry in ccRCC tumor tissues and its association with clinicopathologic characteristics and patient outcome. We further evaluated whether this parameter could add additional prognostic information to well-established pathologic factors and prognostic models.

## Methods

### Patients

A total of 267 patients diagnosed with clear-cell RCC (ccRCC) at Zhongshan Hospital (Shanghai, China) were retrospectively included in the study. We enrolled a training cohort of 195 consecutive patients undergoing nephrectomy between January 2003 and December 2004. For validation, we also enrolled 72 consecutive patients who experienced surgery in 2001. This study was approved by the Ethics Committee of Zhongshan Hospital, Fudan University. Informed consent was obtained from each patient. For each patient, the following clinicopathologic information was collected: age, gender, tumor size, TNM stage, Fuhrman grade, presence of histologic tumor necrosis, and eastern cooperative oncology group performance status (ECOG-PS). Patients were staged using radiographic reports and postoperative pathological data, and were reassigned according to 2010 AJCC TNM classification. None of the patients received neoadjuvant treatment. Patients who died within 30 days of surgery or before discharge were excluded from the study. CSS was calculated from the date of surgery to the date of death or last follow-up, and RFS was calculated from the date of surgery to the date of recurrence or last follow-up. Patients with metastatic disease were not included in the analyses using RFS as the endpoint.

Patients with localized RCC were treated with radical or partial nephrectomy, and patients with metastatic RCC were treated with cytoreductive nephrectomy followed by interferon-α-based immunotherapy. After surgery, patients were evaluated with physical examination, laboratory studies, chest imaging, and abdominal ultrasound or CT scan every six months for the first two years and annually thereafter. Survival status was updated in October 2013. Median follow-up was 103 months (range, 11–120 months) in the training cohort and median follow-up was 72 months (range, 18–118 months) in the validation cohort.

### Tissue microarray (TMA) and immunohistochemistry

Tumor samples were reviewed histologically using hematoxylin and eosin staining, and then we marked representative areas more centrally on the paraffin blocks away from hemorrhagic and necrotic areas. Duplicate 1.0-mm tissue cores from two different areas were used to construct the TMA. Primary antibody against human CSF-1 (Dilution, 1:200; ab52864; Abcam, Cambridge, MA, USA) was used in the procedure. The specificity of the antibody was confirmed by western blot using RCC cell lines. Tissue samples processed similarly, except for the omission of the primary antibody, were used as negative controls in immunohistochemistry. The immunostaining was evaluated by two pathologists (L. Chen and Q. Fu) without the knowledge of patient outcome. A semi-quantitative immunohistochemistry score on a scale of 0–300 was calculated for each sample by multiplying the staining intensity (0, no staining; 1, weak; 2, moderate; and 3, strong) and the percentage of cells (0–100%) at each intensity level [19]. For each patient, the mean score of duplicates was used for statistical analyses [20]. The score agreement between two spots was evaluated by the kappa value, which was excellent (0.82) for CSF-1 expression. The “minimum *P* value” approach was used to obtain the cutoff providing the most optimal separation between the groups of patients in the training cohort related to their CSS by X-tile software. The validation cohort was separated into CSF-1-low patients and CSF-1-high patients with the same cutoff value.

### Statistical analyses

MedCalc 12.7.0. and Stata 12.0. were used to perform statistical analyses. Correlations between immunohistochemical variables and clinicopathologic characteristics were analyzed with χ2 and t tests. Kaplan-Meier method with log-rank test was applied to compare survival curves. All statistical tests were two sided and performed at a significance level of 0.05. Cox regression models were used to analyze the impact of prognostic factors on RFS and CSS. The predictive accuracy of various Cox regression models was quantified by Harrell's concordance index (C-index), which ranges from 0.5 (no predictive power) to 1 (perfect prediction).

## Results

### Patient characteristics and associations with CSF-1 expression

We analyzed a total of 267 patients with ccRCC, 195 in the training cohort and 72 in the validation cohort (Table 1). By comparison, the validation cohort had more patients with early-stage (TNM stage I/II) disease. The two cohorts were well matched for other pathological characteristics. Nine (4.6%) patients had recurrence in the training cohort; fifty four (27.7%) patients died from ccRCC during the follow-up period. In the validation cohort, eight (11.1%) patients had recurrence; twenty four (33.3%) patients died from ccRCC at the time of last follow-up.

**Table 1:** **Patient characteristics and associations with CSF-1 expression**

	Training cohort					Validation cohort				
Variable	Patients		CSF-1 expression		*P*	Patients		CSF-1 expression		*P*
	Number	%	Low (n = 99)	High (n = 96)		Number	%	Low (n = 43)	High (n = 29)	
Age (years)*	55.3		56.2	54.3	0.245	59.8		61.5	59.1	0.421
Gender					0.987					0.982
Male	137	70.3	70	67		51	70.8	31	20	
Female	58	29.7	29	29		21	29.3	12	9	
Tumor size (cm)*	4.7		4.4	4.9	0.194	5.2		4.8	5.8	0.106
TNM stage					0.091					0.077
I + II	134	68.7	74	60		56	77.8	37	19	
III + IV	61	31.3	25	36		16	22.2	6	10	
Fuhrman grade					**0.001**					**0.007**
1 + 2	122	62.6	74	48		49	68.1	35	14	
3 + 4	73	37.4	25	48		23	31.9	8	15	
Necrosis					**0.013**					**0.039**
Absent	150	76.9	84	66		55	76.4	37	18	
Present	45	23.1	15	30		17	23.6	6	11	
ECOG PS										
0	160	82.1	80	80	0.785	59	81.9	36	23	0.869
≥1	35	17.9	19	16		13	18.1	7	6	

CSF-1 = Colony Stimulating Factor 1.

ECOG-PS = Eastern Cooperative Oncology Group performance status.

*Student’s t test and χ2 test for all the other analyses.

The bold characters indicate that these *P* values are considered statistically significant.

**Figure 1:**
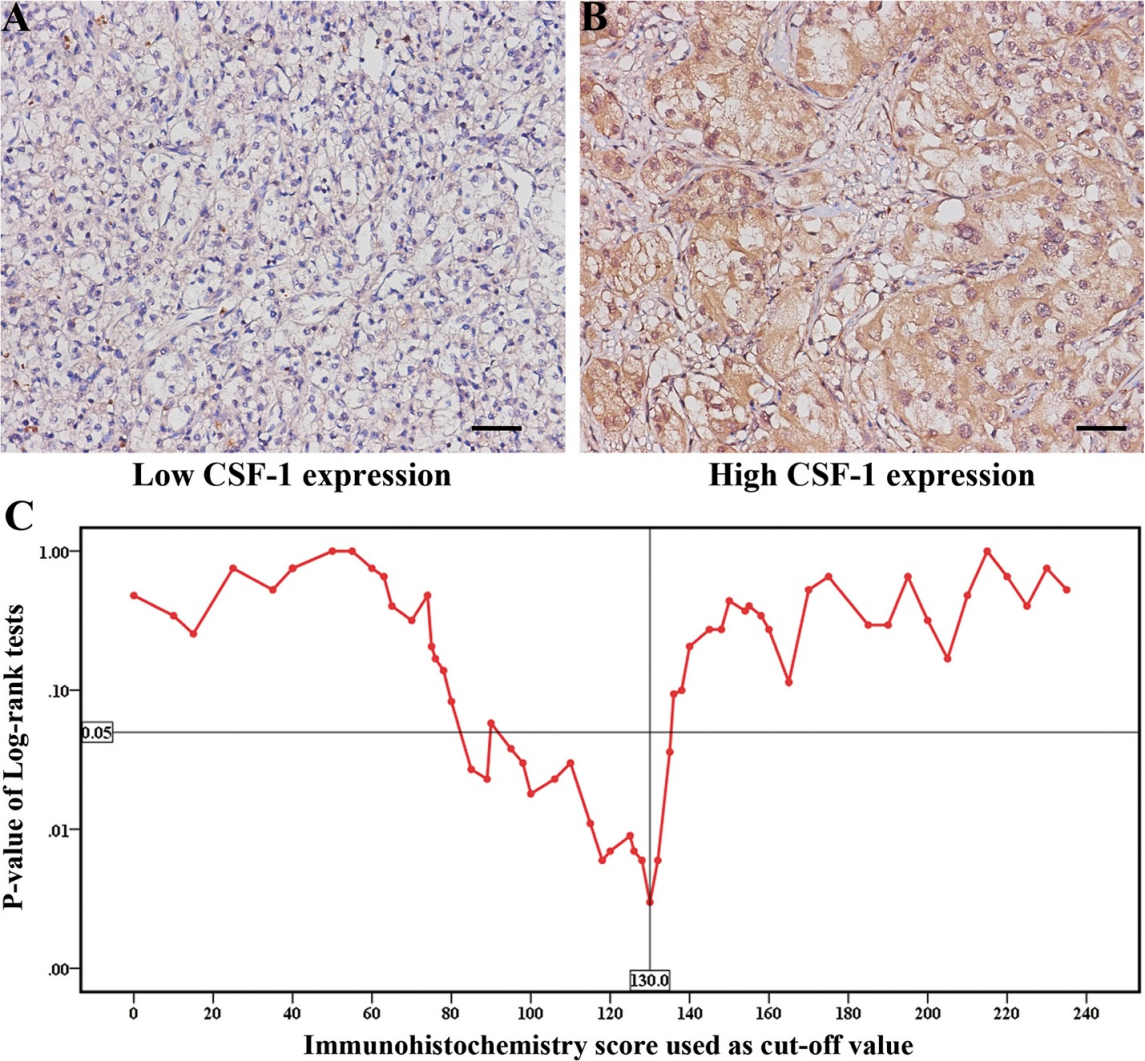
**CSF-1 expression in ccRCC tissues and the result of “minimum*P*value” approach. (A,B)** Representative CSF-1 immunohistochemical images of **(A)** low expression (score = 15) and **(B)** high expression (score = 240), respectively. Scale bar, 50 μm (original magnification × 200). **(C)** The result of “minimum *P* value” approach and 130 had the best discriminatory power.

**Figure 2:**
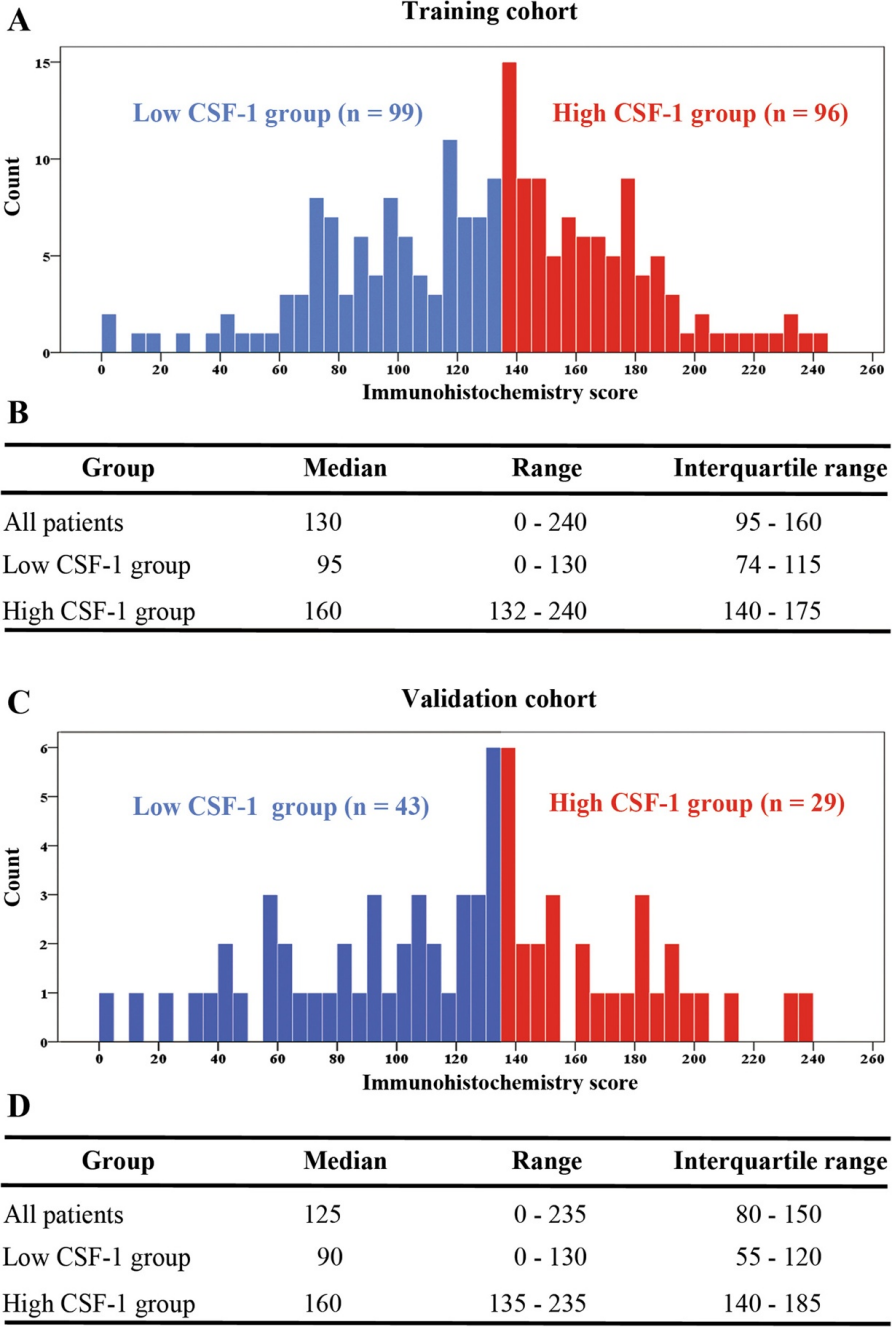
**The descriptive statistics of immunohistochemistry score data in two independent cohorts. (A,B)** The descriptive statistics of immunohistochemistry score of all patients and low/high-CSF-1 expression subgroups in the training cohort. **(C,D)** The descriptive statistics of immunohistochemistry score of all patients and low/high-CSF-1 expression subgroups in the validation cohort.

CSF-1 positive staining mainly appeared in the cytoplasm of tumor cells. Representative CSF-1 immunohistochemical images of low expression (score = 15) and high expression (score = 240) have been shown in Figure 1A and B, respectively. According to the result from the “minimum *P* value” approach (Figure 1C), 130 was determined as the cutoff immunohistochemistry score with the best discriminatory power, which separated the training cohort into low CSF-1 group (99 patients) and high CSF-1 group (96 patients). The validation cohort was separated into low CSF-1 group (43 patients) and high CSF-1 group (29 patients) with the same cutoff value. The descriptive statistics of immunohistochemistry score of all patients and low/high-CSF-1 expression subgroups in the training cohort have been presented in Figure 2A and B, and that of validation cohort was shown in Figure 2C and D. Correlations between CSF-1 expression and clinicopathologic features are summarized in Table 1. CSF-1 expression was positively correlated with Fuhrman grade (*P* = 0.001 in the training cohort and *P* = 0.007 in the validation cohort) and tumor necrosis (*P* = 0.013 in the training cohort and *P* = 0.039 in the validation cohort).

### High CSF-1 expression is associated with poor prognosis

**Figure 3:**
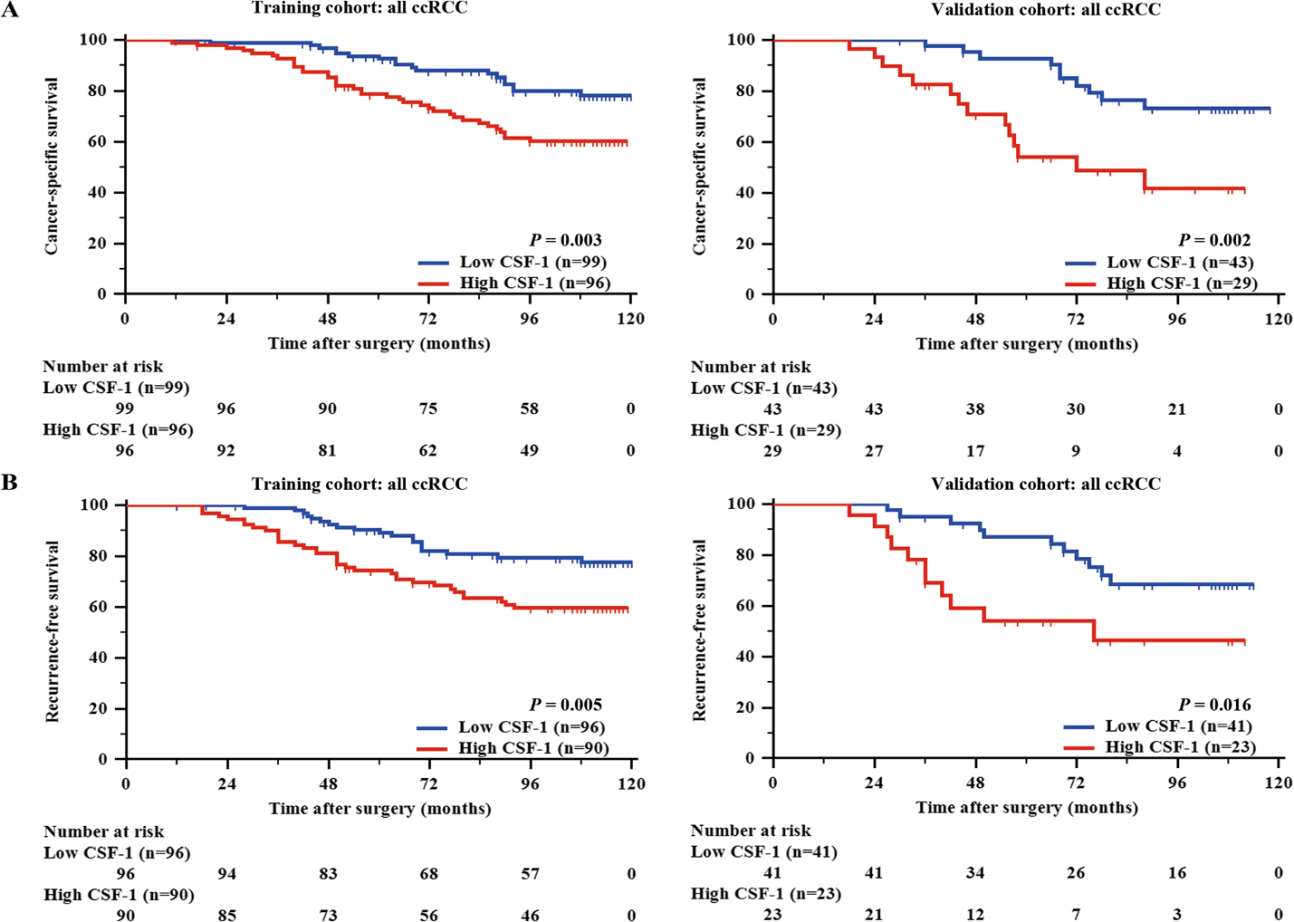
**Kaplan-Meier analyses for CSS and RFS of all patients with ccRCC. (A,B)** Kaplan-Meier analyses for CSS and RFS of ccRCC patients according to CSF-1 expression in all patients **(A)** CSS (left, training cohort, n = 195, *P* = 0.003; right, validation cohort, n = 72, *P* = 0.002), **(B)** RFS (left, training cohort, n = 186, *P* = 0.005; right, validation cohort, n = 64, *P* = 0.016).

**Figure 4:**
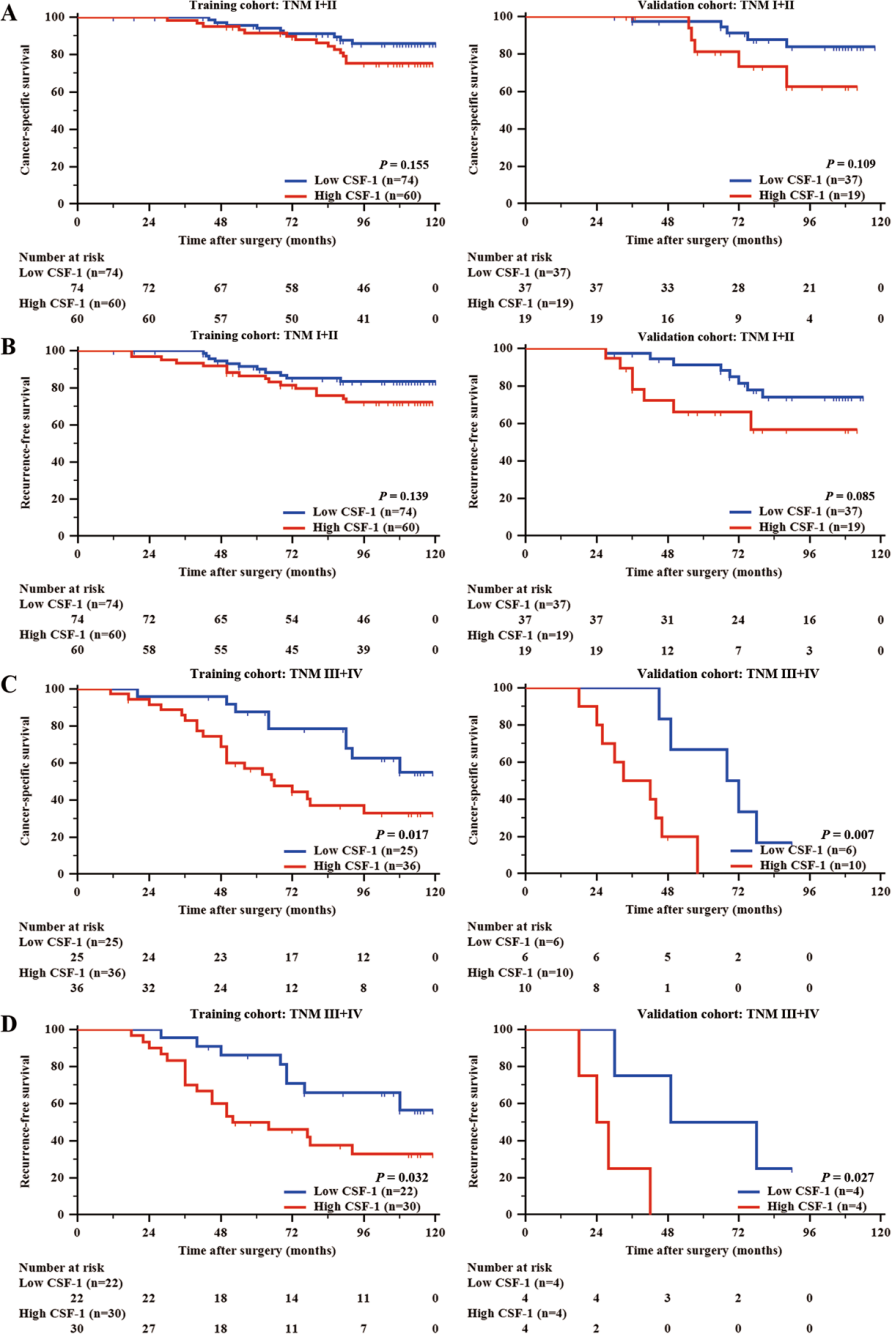
**Kaplan-Meier analyses for CSS and RFS of patients with ccRCC in TNM subgroups. (A,B)** Kaplan-Meier analyses for CSS and RFS of ccRCC patients according to CSF-1 expression in TNM I + II **(A)** CSS (left, training cohort, n = 134, *P* = 0.155; right, validation cohort, n = 56, *P* = 0.109) **(B)** RFS (left, training cohort, n = 134, *P* = 0.139; right, validation cohort, n = 56, *P* = 0.085). **(C,D)** Kaplan-Meier analyses for CSS and RFS of ccRCC patients according to CSF-1 expression in patients of TNM III + IV **(C)** CSS (left, training cohort, n = 61, *P* = 0.017; right, validation cohort, n = 16, *P* = 0.007) **(D)** RFS (left, training cohort, n = 52, *P* = 0.032; right, validation cohort, n = 8, *P* = 0.027).

As shown in Figure 3A and B, Kaplan-Meier survival analyses indicated that high CSF-1 expression was associated with shorter CSS and RFS in the training cohort (*P* = 0.003 and *P* = 0.005, respectively). We next evaluated the independent prognostic value of CSF-1 expression using Cox regression analysis (Table 2). With adjustment for other known pathologic predictors of patient outcome, CSF-1 expression was proven to be independently predictive of CSS (HR 2.609, 95% CI 1.432-4.755, *P* = 0.002 for the training cohort; HR 4.435, 95% CI 1.478-13.308, *P* = 0.008 for the validation cohort) and RFS (HR 2.075, 95% CI 1.168-3.687, *P* = 0.013 for the training cohort; HR 3.460, 95% CI 1.328-9.012, *P* = 0.012 for the validation cohort) for patients with ccRCC after surgery in both cohorts. We further performed a subgroup analysis by TNM stage. The prognostic value of CSF-1 expression was restricted to patients with TNM stage III/IV disease (Figures 4C and D). In contrast, the patients with TNM stage I/II could not be stratified by CSF-1 expression (Figure 4A and B). These results were replicated in our validation cohort (Figure 4).

**Table 2:** **Univariate and multivariate cox regression analyses in the two independent cohorts**

Characteristic	Training cohort			Validation cohort		
	Univariate*P*	Multivariate		Univariate*P*	Multivariate	
		HR (95% CI)	*P*		HR (95% CI)	*P*
**Cancer-specific survival**						
Age (years)	0.276			0.136		
Gender (male vs female)	0.929			0.138		
Tumor size (cm)	<0.001	1.071(0.973-1.180)	0.163	0.001	1.008(0.850-1.195)	0.932
TNM stage (III + IV vs I + II)	<0.001	3.847(2.195-6.743)	<0.001	<0.001	18.197(6.053-54.701)	<0.001
Fuhrman grade (3 + 4 vs 1 + 2)	0.001	2.308(1.342-3.970)	0.003	<0.001	3.648(1.314-10.126)	0.014
Necrosis (present vs absent)	0.015	1.183(0.657-2.127)	0.578	0.014	1.270(0.505-3.197)	0.614
ECOG PS (≥1 vs 0)	<0.001	2.750(1.496-5.056)	0.001	<0.001	7.059(2.233-22.311)	0.001
CSF-1 (high vs low)	0.004	2.609(1.432-4.755)	0.002	0.004	4.435(1.478-13.308)	0.008
**Recurrence-free survival**						
Age (years)	0.113			0.661		
Gender (male vs female)	0.972			0.726		
Tumor size (cm)	0.001	1.081(0.981-1.191)	0.118	0.001	1.154(0.964-1.381)	0.121
TNM stage (III + IV vs I + II)	<0.001	3.095(1.779-5.383)	<0.001	<0.001	10.053(3.198-31.602)	<0.001
Fuhrman grade (3 + 4 vs 1 + 2)	0.002	2.196(1.282-3.760)	0.004	<0.001	2.957(1.197-7.306)	0.019
Necrosis (present vs absent)	0.012	1.180(0.649-2.145)	0.590	0.010	1.156(0.447-2.990)	1.156
ECOG PS (≥1 vs 0)	0.001	2.049(1.082-3.878)	0.028	0.037	5.103(1.494-17.428)	0.010
CSF-1 (high vs low)	0.006	2.075(1.168-3.687)	0.013	0.021	3.460(1.328-9.012)	0.012

HR = Hazard Ratio; 95% CI, 95% confidence interval.

### Extension of established prognostic models with CSF-1 expression

**Figure 5:**
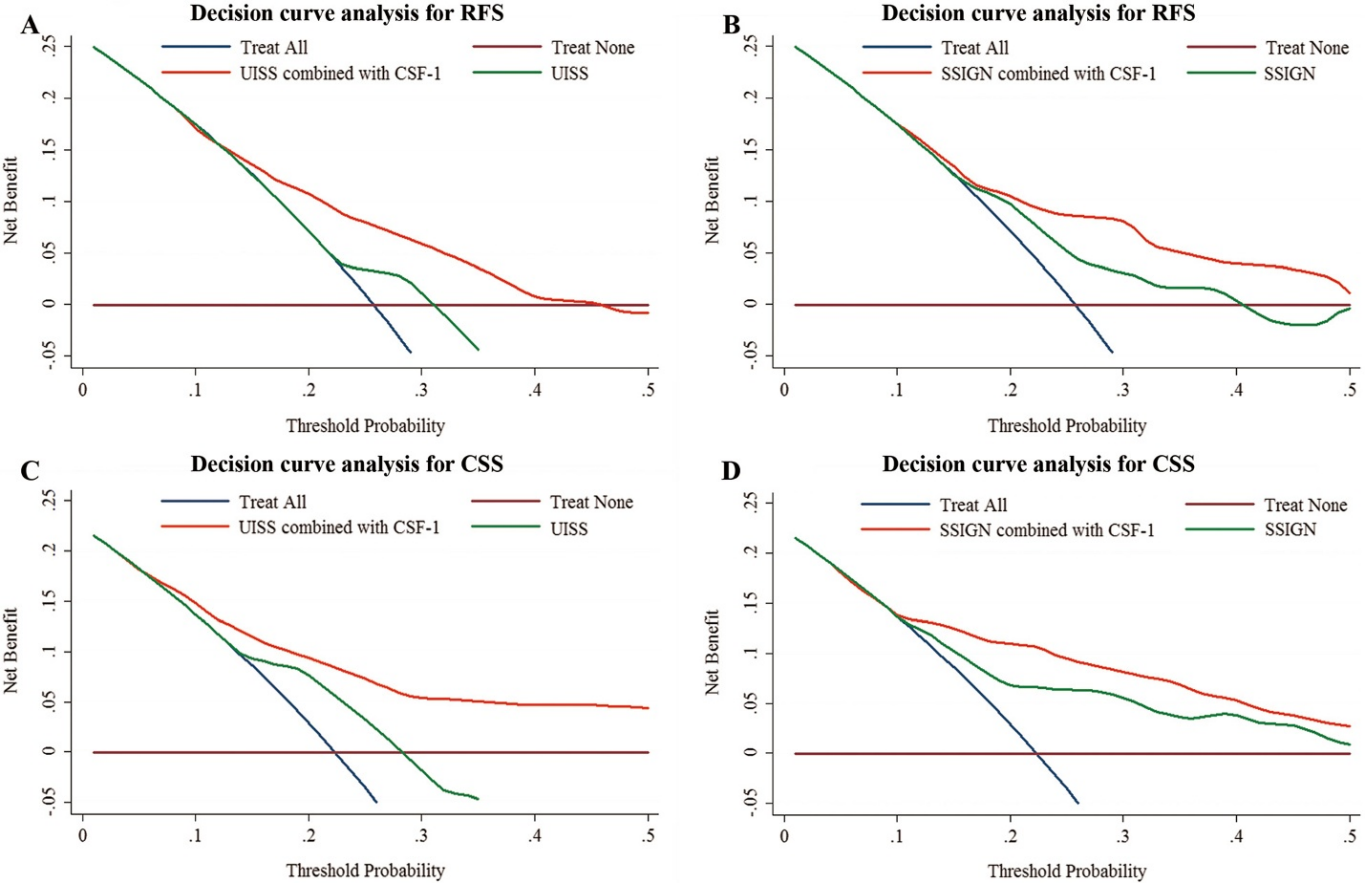
**Comparison of the predictive accuracies of prognostic models with or without CSF-1 expression by decision curve analysis (DCA). (A,B)** DCA of the predictive accuracies of **(A)** UISS and **(B)** SSIGN for predicting RFS; **(C,D)** DCA of the predictive accuracies of **(C)** UISS and **(D)** SSIGN for predicting CSS.

In addition to TNM stage, the UISS and SSIGN scores are often used to determine prognosis and treatment. Then we investigated whether incorporation of CSF-1 expression into these two models would improve their predictive accuracy. Decision curve analysis (DCA) was first performed to compare predictive accuracy of the prognostic models. For RFS (Figure 5A and B), both UISS and SSIGN had a higher net benefit when CSF-1 expression was added. Similar results were found for CSS, the net benefit of UISS and SSIGN was improved after the incorporation of CSF-1 expression (Figure 5C and D).

Then the C-indices of prognostic models with or without CSF-1 expression were calculated (Table 3). For RFS, the C-index of the UISS was improved from 0.638 to 0.678 when CSF-1 expression was added, which was statistically significant (*P* = 0.004). However, the C-index of the SSIGN was slightly increased from 0.710 to 0.718 after the addition of CSF-1, which failed to reach statistical significance (*P* = 0.393). Similarly for CSS, the C-index of the UISS was improved from 0.708 to 0.742 (*P* = 0.001) when CSF-1 expression was supplemented, whereas the C-index of the SSIGN was merely increased from 0.753 to 0.764 (*P* = 0.231) after the incorporation of CSF-1. We further calculated the C-indices with respect to predictive models within TNM stage I/II and III/IV disease, respectively, and the predictive accuracy of the UISS and SSIGN were significantly improved when CSF-1 expression was added only for CSS in TNM stage III/IV subgroup (Table 3).

**Table 3:** **Comparison of the predictive accuracies of prognostic models**

Model	CSS		RFS	
	C-index*	*P* ^†^	C-index*	*P* ^†^
**All patients**				
CSF-1	0.620		0.607	
UISS	0.708		0.638	
UISS combined with CSF-1	0.742	**0.001**	0.678	**0.004**
SSIGN	0.753		0.710	
SSIGN combined with CSF-1	0.764	0.231	0.718	0.393
**TNM stage I/II**				
CSF-1	0.581		0.578	
UISS	0.615		0.619	
UISS combined with CSF-1	0.640	0.188	0.642	0.224
SSIGN	0.647		0.665	
SSIGN combined with CSF-1	0.656	0.821	0.669	0.652
**TNM stage III/IV**				
CSF-1	0.631		0.623	
UISS	0.633		0.524	
UISS combined with CSF-1	0.721	**0.004**	0.625	0.079
SSIGN	0.695		0.624	
SSIGN combined with CSF-1	0.754	**0.012**	0.693	0.060

*A larger C-index represents a better discriminatory power.

^†^Compared with the original model without CSF-1 expression.

The bold characters indicate that these *P* values are considered statistically significant.

## Discussion

In this study, we demonstrated that high CSF-1 expression is a predictor of poor prognosis for surgically treated ccRCC patients. Moreover, the prognostic value of CSF-1 was restricted to patients with stage III/IV disease. When incorporated into well-established prognostic models, CSF-1 expression could significantly improve the predictive accuracy of UISS.

CSF-1 is a secreted cytokine impacting the differentiation of hematopoietic stem cells into macrophages. The pleiotrophic actions of CSF-1 are transduced by its sole receptor CSF-1R [21]. As the most abundant tumor-infiltrating immune cells, tumor associated macrophages (TAM) are significant for fostering tumor progression. TAM display diversely polarized programs comprising proinflammatory M1 macrophages and immunosuppressive M2 macrophages. CSF-1 has been demonstrated as a mediator polarizing macrophages into an M2 phenotype which can promote tumor-induced immunosuppression in established tumors [7,8]. Previous studies have revealed that both high CSF-1 expression and high macrophages density were associated with disease progression and poor survival in several malignancies, such as liver and prostate cancers, which suggests that high CSF-1 expression might be associated with more inflammatory cell infiltration [20,22-27]. Additionally, Menke et al. further stated that CSF-1 and CSF-1R expression were associated with infiltrating macrophages in RCC and adjacent TEC, indicating that the magnitude of CSF-1 and CSF-1R is an index of the extent of macrophages [28]. Inflammatory infiltration might be different between high and low CSF-1 expression subjects, which merits further investigation in our next research to reveal the specific roles of CSF-1 in malignant transformation of ccRCC. In RCC, apart from polarizing macrophages into an M2 phenotype, CSF-1 could also lead to the activation of signal transducer and activator of transcription-3 (Stat3) which promotes cell survival and proliferation as well as immune responses associated with tumor progression [17]. Similar results were obtained in breast, ovarian and lung cancers where a CSF-1 dependent autocrine loop contributes to tumor invasiveness and metastasis [28-31].

The natural history of RCC is complex and influenced by factors other than pathologic stage. Therefore, integrated prognostic algorithms are needed to better predict patient outcomes. Currently, UISS and SSIGN scores are widely used predictive models to identify patients at a greater risk of disease progression after surgery. However, these models only focus on the characteristics of tumor cells, but ignore the components of tumor microenvironment which also plays an important role in tumor development and progression. Therefore, it is reasonable that incorporation of CSF-1 expression into established predictive models would improve prognostic stratification. The predictive accuracy of the UISS was improved when CSF-1 expression was added, which was statistically significant for RFS and CSS. However, the predictive accuracy of the SSIGN was slightly increased after the addition of CSF-1, which failed to reach statistical significance for RFS and CSS. Collectively, these results indicated that incorporation of CSF-1 expression could significantly improve the predictive accuracy of UISS, but not SSIGN. According to Parkers, it is better to utilize tumor-based prognostic biomarkers in a sequential or stepwise manner [32]. In other words, instead of immutably integrating CSF-1 expression into an existing prognostic model, we support its use on an as-needed basis. Oncologists or urologists could first determine prognosis for a RCC patient using conventional pathologic factors or prognostic models. After that, prognostic information maybe further refined by biomarker testing if physicians and patients think it is necessary. This information is useful in selecting patients for additional treatment and customizing postsurgical surveillance.

There are several limitations of our study that warrant further discussion. Firstly, our findings need to be replicated and externally validated in an independent cohort. Secondly, the immunohistochemistry analysis is always somewhat subjective. To minimize this impact in our study, duplicate tissue cores from the same tumor were used to construct the tissue microarray, highly standardized IHC protocols were applied, and two experienced urologic pathologists blinded to the clinical data evaluated immunostained slides. Thirdly, to facilitate graphical presentation (Kaplan-Meier curves) and potential clinical use, CSF-1 expression measured as a continuous variable was dichotomized into low and high groups at the cost of great information loss. Furthermore, determination of cases with CSF-1 expression near cutoff value could be difficult because a difference of 20–40 in the semiquantitative immunohistochemistry assessment could be quite subjective, especially in the clinical setting. Fourthly, functional studies are needed to elucidate the biological mechanisms involved in this association.

## Conclusion

In conclusion, the present study demonstrated that CSF-1 expression is an independent adverse prognostic biomarker for recurrence and survival of patients with ccRCC after nephrectomy. Incorporating CSF-1 expression into the UISS prognostic model could significantly improve its predictive accuracy.

## Abbreviations

CSF-1: Colony stimulating factor-1
RCC: Renal cell carcinoma
ccRCC: Clear-cell renal cell carcinoma
CSS: Cancer-specific survival
RFS: Recurrence-free survival
C-index: Harrell's concordance index
UISS: University of California Los Angeles Integrated Staging System
SSIGN: Mayo stage, size, grade, and necrosis score
M-CSF: Macrophage colony-stimulating factor
CSF-1R: Colony stimulating factor-1 receptor
ECOG-PS: Eastern cooperative oncology group performance status
TMA: Tissue microarray
TAM: Tumor associated macrophages
Stat3: Signal transducer and activator of transcription-3
DCA: Decision curve analysis
